# How linear features alter predator movement and the functional response

**DOI:** 10.1098/rsfs.2011.0086

**Published:** 2012-01-18

**Authors:** Hannah W. McKenzie, Evelyn H. Merrill, Raymond J. Spiteri, Mark A. Lewis

**Affiliations:** 1 Centre for Mathematical Biology, Department of Mathematical and Statistical Sciences, 632 CAB, University of Alberta, Edmonton, Alberta, Canada T6G 2G1; 2 Department of Biological Sciences, CW 405, Biological Sciences Building, University of Alberta, Edmonton, Alberta, Canada T6G 2E9; 3 Department of Computer Science, 176 Thorvaldson Building, University of Saskatchewan, 110 Science Place, Saskatoon, Saskatchewan, Canada S7N 5C9

**Keywords:** encounter rate, mean first passage time, seismic lines, spatial heterogeneity, wolf movement

## Abstract

In areas of oil and gas exploration, seismic lines have been reported to alter the movement patterns of wolves (*Canis lupus*). We developed a mechanistic first passage time model, based on an anisotropic elliptic partial differential equation, and used this to explore how wolf movement responses to seismic lines influence the encounter rate of the wolves with their prey. The model was parametrized using 5 min GPS location data. These data showed that wolves travelled faster on seismic lines and had a higher probability of staying on a seismic line once they were on it. We simulated wolf movement on a range of seismic line densities and drew implications for the rate of predator–prey interactions as described by the functional response. The functional response exhibited a more than linear increase with respect to prey density (type III) as well as interactions with seismic line density. Encounter rates were significantly higher in landscapes with high seismic line density and were most pronounced at low prey densities. This suggests that prey at low population densities are at higher risk in environments with a high seismic line density unless they learn to avoid them.

## 1. Introduction

One of the most common functional response models is the Holling disc equation [1]. In the disc equation, the encounter rate is assumed to be linearly related to prey density by the ‘instantaneous search rate’ [2] or ‘area of discovery’ [1]. This leads to the type I functional response, or the type II functional response once handling time is included. Previous theoretical work suggests that this assumption of linearity may not hold if predator movement is partially random, i.e. the new area searched by the predator per unit time is not constant [3]. The results suggest that, in one dimension and under certain assumptions regarding prey availability, the encounter rate of predators undergoing a random walk has a quadratic relationship with prey density. This quadratic relationship arises because during a random walk, predators may repeatedly return to regions previously searched. When substituted into the disc equation, McKenzie *et al.* [3] showed that a quadratic encounter rate leads to a type III functional response, thereby demonstrating a link between predator movement modes and the shape of the functional response. McKenzie *et al.* [3] worked in a simplified theoretical framework, but it is known that predators searching for prey in spatially heterogeneous habitats have both directed and random components to their movement. In this case, the encounter rate is expected to be a more complex function of prey density, perhaps involving both linear and quadratic terms, and therefore possibly leading to some combination of the familiar type II and type III functional responses.

Owing to the challenges of reproducing the theoretical arguments used by McKenzie *et al.* [3] under assumptions of more realistic predator movement in heterogeneous landscapes, here we take a different approach to investigate the link between predator movement and the functional response. We first derive more realistic predator movement models based on predator movement data. Then, using these movement models, we simulate prey encounter rates over a range of prey densities and fit the simulated encounter rate data to the encounter rate models. Finally, we substitute the encounter rate function into the disc equation to determine the shape of the resulting functional response.

For the source of spatial heterogeneity, we focus on seismic lines, which are narrow, linear stretches of forest, cleared for energy exploration ([4] and electronic supplementary material, appendix figure S1*a*). Wolves (*Canis lupus*) have been found to both avoid and use linear features, depending on the linear feature density and level of human use [5–8]. Because studies have shown that wolves moved up to 2.8 times faster on linear features than in the forest [6], it has been hypothesized that seismic lines may benefit wolves if increased travel rates result in higher encounter rates with prey [9–11]. Consequently, in landscapes with high densities of seismic lines, species such as caribou (*Rangifer tarandus*) and elk (*Cervus elaphus*) may be at higher predation risk owing to higher encounter rates [12–14].

In this paper, we use this example of wolf movement in response to seismic lines to demonstrate the link between animal response to spatial heterogeneity and functional response models. We follow the mean first passage time approach described McKenzie *et al.* [3] extended to two dimensions. In the context of predation, we define the mean first passage time as the average time required for a moving predator to locate a first stationary prey, given a specific prey density [3]. Therefore, the inverse of mean first passage time is the encounter rate at that prey density. Using this framework, it is possible to formulate first passage time models that reflect the effect of landscape features on predator movement. After evaluating the influence of different movement responses of wolves on seismic lines, we use model outputs to assess a set of *a priori* models relating encounter rate to seismic line and prey density, both independently and together. The functional forms of the candidate models are chosen to reflect different underlying predator movement mechanisms. Using the best-fit model, we investigate the implications for wolf functional responses owing to increased predator mobility in the presence of seismic lines, and discuss how these ideas could be extended to include other sources of landscape heterogeneity.

## 2. Methods

### 2.1. Modelling

Encounter rate is the rate at which predators encounter prey in the landscape (unit: time^−1^). Here, we consider a mechanistic model for encounter rate that includes the effect of predator movement. We modelled encounter rate using mean first passage time. Mean first passage time, *T*(**x**) (unit: time), is the average time required for a predator starting at location **x** to encounter any number of stationary prey, given a specified prey distribution and landscape. Although it would be possible to approximate *T*(**x**) from averaging repeated random walk simulations from each point **x** of interest, an alternative, computationally efficient approach uses a partial differential equation to describe the surface *T*(**x**). This approach is based on the Fokker–Planck approximation for animal movement patterns that is described in depth by Turchin [15] and in particular for this model by McKenzie *et al.* [3]. To derive a partial differential equation for *T*, McKenzie *et al.* [3] begin by encoding aspects of wolf movement believed to be important in determining space use, in this case focusing on wolf response to seismic lines, into a set of probabilistic movement rules for individual wolves. These are then translated, using mathematical approximations, into the following partial differential equation for *T*.2.1
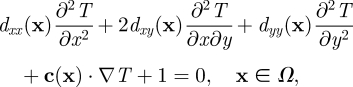
where the domain *Ω* is the landscape of interest. In this mathematical formulation, as is the case in the Holling disc equation, the prey are assumed to be stationary and are represented by interior Dirichlet (absorbing disc) boundary conditions. This means that if the predator starts within perception radius *r* of a prey item, the time required to locate a prey item, *T*, equals zero. Defining prey in this way allows us to study the behaviour of the first passage time model for any finite number of prey, with spatial locations of our choosing. The particular prey scenarios we considered are further described in the §2.3. The coefficients of the partial derivatives are derived mechanistically based on the underlying predator movement behaviour (electronic supplementary material, appendix). Equation (2.1) includes diffusive movement in the first three terms (*d*_*xx*_, *d*_*xy*_ and *d*_*yy*_), and advective movement in the fourth term (**c**), which together approximate animal movement. The dependence of the diffusion and advection terms on the location **x** indicates that movement terms can vary from one location to other. Directionality in the movement terms can arise either from the advection term (**c**), which indicates a directional bias in the movement, or from anisotropic diffusion (unequal values for *d*_*xx*_, *d*_*xy*_ and *d*_*yy*_), which indicates different levels of random movement in different directions. For example, in the absence of other influences, far from the landscape features predators may move in a random fashion. However, their movement may become more directed as they interact with landscape features.

The solution to equation (2.1) is a two-dimensional surface *T*(**x**), where the value of the surface at each point in space is the mean first passage time of a predator located at **x**. Therefore, the value of the surface at each point in space indicates how long, on average, a predator starting **x** would need to search before locating a prey item. This surface provides a picture of how the mean first passage time varies in space, and can be summarized by the spatially averaged mean first passage time,2.2
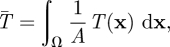
where *A* is the area of *Ω*. The average mean first passage time is the mean first passage time assuming that the predator initially is randomly distributed in the landscape. For a given landscape and prey density, the average mean first passage time can then be related to the encounter rate by2.3
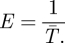
It would be possible to account for a non-random initial distribution of the predator by replacing the uniform weight 1/*A* in equation (2.2) with a more general probability density function for the initial location of the predator *u*(**x**). For example, the distribution *u*(**x**) could be a statistical home range model, such as that of Kernohan *et al.* [16], or a mechanistic home range model, such as that of Moorcroft & Lewis [17].

Based on previous studies of wolf movement in landscapes with linear features [6,7], wolves are likely to show varied responses to these features. In the simplest model (no response), wolves do not alter their movement in response to seismic lines. This model is analogous to wolf movement in a landscape without seismic lines and corresponds to a random walk everywhere in the landscape. Although this model may be biologically unreasonable [5,8], it provides a baseline for comparison in understanding the effect of increasing prey density on encounter rate in the absence of seismic lines. In the second model (anisotropic diffusion), wolves move faster on seismic lines and are more likely to continue along them in either direction once on them. Mechanistically, we considered the anisotropy as arising from higher movement speed in either direction along the seismic lines as well as possible correlations in the random walk when moving along the seismic lines. Both these mechanisms individually would lead to an enhanced diffusion coefficient [18] but only in the direction of the seismic line. This was incorporated mathematically by allowing anisotropic diffusion on seismic lines, where diffusion was increased along the seismic line and decreased across it. This model corresponds to a random walk away from seismic lines and a random walk with anisotropic diffusion on seismic lines. The final model (anisotropic diffusion + bias) was an extension of the previous model where, in addition to being more likely to continue along seismic lines when on them, wolves also biased their movement towards the seismic line when near them. This model corresponds to a random walk far from seismic lines, a biased random walk near seismic lines, and a random walk with anisotropic diffusion on seismic lines. In addition to the terms previously discussed, the equation for this model also includes an advection term in regions near seismic lines, pointing in the direction towards the nearest seismic line. Each of the three wolf movement models leads to different forms of equation (2.1) as summarized in table 1. The details underlying the calculation of the coefficients are given in the electronic supplementary material, appendix.

**Table 1.** Summary of the proposed wolf movement models and the corresponding form of the mean first passage time equation for each. Exact formulae for the coefficients are given in the electronic supplementary material, appendix.

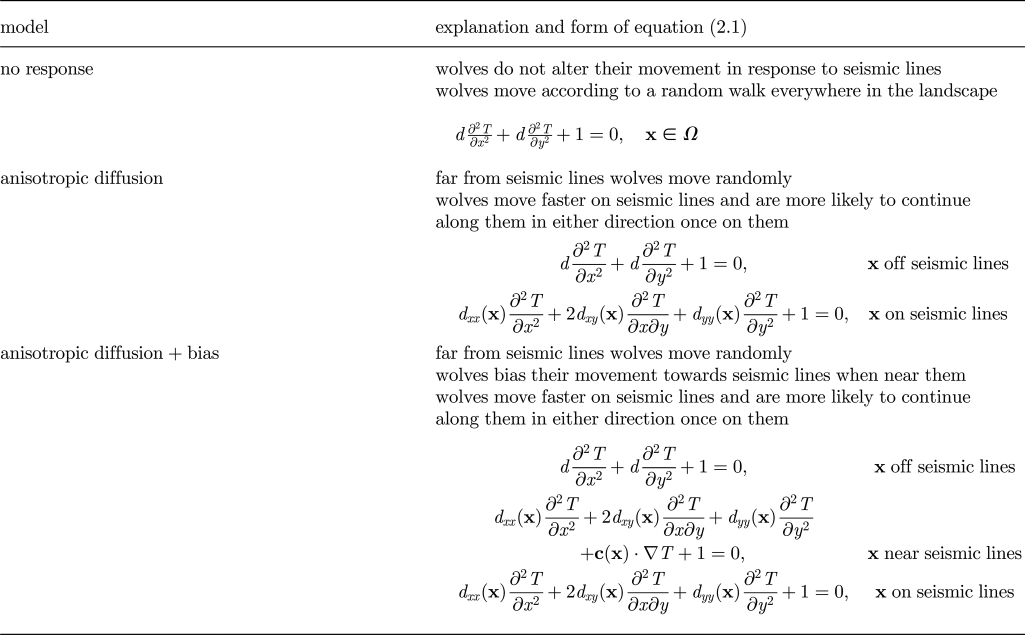

### 2.2. Wolf movements

We studied the movements of four GPS-collared wolves in the central east slopes of the Rocky Mountains, Alberta, Canada where average daily mean temperatures were −7.5°C in winter (January/February) and 1°C in spring (March/April) and total snowfall was 54 cm in winter and 24 cm in spring. This area supports prey populations of moose (*Alces alces*), mule and white-tailed deer (*Odocoilues hemionus* and *Odocoiles virginianus*), and elk (*Cervus canadensis*), as well as their main predator, wolves. Wolf densities ranged from 9.7 to 22.3 wolves per 1000 km^2^ [19]. Seismic line density varied from 0.18 km km^−2^ near the Western border to 4.4 km km^−2^ near Rocky Mountain House, with a mean of 1.8 km km^−2^. It is likely that seismic lines experienced a range of human use year-round for hunting, trapping, snowmobiling, off-roading and hiking.

Movement data were obtained from GPS locations of collared wolves from four individuals in three packs during January–April 2005, occupying territories across a gradient of seismic line densities from 1.73 to 3.60 km km^−2^ [20]. Collars were programmed to collect locations at 5 min intervals and successfully recorded locations on 90 per cent of fixed attempts. Data were downloaded upon retrieval via a remote release mechanism (three wolves) or recapture (one wolf). Wolves were considered to be independent units because they were either from different packs or the data were collected during different time periods.

We described movements via vectors joining the current and next consecutive wolf location. Each vector was characterized by step length and movement direction (with direction North having 0°). To investigate the appropriateness of a diffusion-type model for movement, we calculated the mean-squared displacement for each wolf for time intervals of length 5, 15, 30, 60 and 120 min. A simple diffusion model with no correlations predicts a linear increase in the mean square displacement (m.s.d.) as a function of time interval. We also calculated correlations in successive movement directions using the circular correlation coefficient (*ρ*_aa_)_s_ [21].

Our movement model characterized wolf movement via step length (*ρ* > 0) and *relative* move direction. Relative move direction (−180° ≤ *ξ* ≤ 180° ) is the angle between the ‘beeline’ move direction of the animal and the direction of a straight line pointing from the current location towards the nearest seismic line (electronic supplementary material, appendix figure S1). A relative move direction of *ξ* ± 90° represents moves along the seismic line *ξ* = 0° represents moves towards the seismic line, and *ξ* ± 180° represents moves away from the seismic line. Because GPS measurement error may result in incorrect inference of move direction between locations that are less than 5 s.d. of the GPS error kernel apart [22], we considered only those relative move directions with corresponding move distances greater than 55 m [23]. However, we used all of the distances when calculating step lengths so as to avoid introducing a bias towards longer moves.

To understand how the distance of a wolf to a seismic line affected the step lengths and relative move directions of the wolves, we classified wolf locations into three groups: on, near and off seismic lines. Seismic lines were assumed to have an average width of 5 m and were buffered by an additional 24.5 m on each side to account for GPS measurement error in wolf locations [23]. Locations within the GPS error buffer were classified as ‘on’. Locations between the GPS error buffer and the distance at which we assumed wolves perceived seismic lines were classified as ‘near’. We arbitrarily chose a distance of 50 m to represent the distance at which seismic lines might be visible to wolves. Locations beyond the perceptual range were classified as ‘off’. We quantified and compared the distributions of each near, far and off seismic lines.

Step lengths of canids often follow the exponential distribution2.4
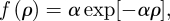
where *α* is the mean step length [17]. The maximum-likelihood estimate 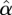 is the sample mean, 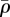. We compared the mean step lengths on, near and off seismic lines using 90% confidence intervals obtained by non-parametric bootstrapping [24].

The von Mises distribution is commonly used to describe animal movement directions ([17] and electronic supplementary material, appendix). We assumed that the distribution of relative move directions of wolves on seismic lines followed the bivariate von Mises distribution2.5
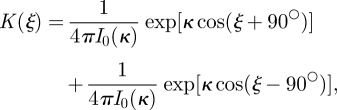
where the movement directions are oriented in the direction of the seismic line. To determine whether anisotropic movement was present, we tested the null hypothesis *H*_0_ : *κ* = 0 (no tendency to continue along the seismic line in the next move) against the alternative hypothesis *H*_*a*_ : *κ* > 0 (tendency to continue along the seismic line in the next move) using the parametric bootstrap likelihood-ratio (PBLR) test [25,26]. For moves near seismic lines, we assumed that the distribution of relative move directions was univariate von Mises,2.6
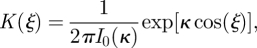
where the movement directions are oriented towards the seismic line. To determine whether bias towards the lines was present, we tested the null hypothesis *H*_0_ : *κ* = 0 (no tendency to move towards the seismic line in the next move) against the alternative hypothesis *H*_0_ : *κ* > 0 (tendency to move towards the seismic line in the next move) using the PBLR test [25,26]. In each case, the maximum-likelihood estimate 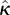 was found by numerical maximization of the likelihood function and non-parametric bootstrapped 90% confidence intervals constructed [24].

To investigate the effect that possible correlations in successive movement directions would have on the movement patterns, we simulated 1000 wolf movement paths based on choosing the turning angle and step length randomly from the measured data values. These simulations were repeated for each individual wolf for situations on and off linear features and the mean-squared displacement per day was calculated as a summary statistic.

### 2.3. Model scenarios

To study the effect of seismic line density and prey density on encounter rate for wolves, we solved equation (2.1) numerically for various scenarios using COMSOL Multiphysics (COMSOL, Inc. Stockholm, Sweden). We chose the domain *Ω* to be a 25 km × 25 km landscape, similar in area to the average home range size of the wolves in the study area [19]. The edges of the domain were subject to Neumann (reflecting) boundary conditions. This corresponds to the wolf remaining within its home range. We then computed the encounter rate for each scenario using equation (2.3). Because wolves remain at a kill site for several hours or longer and typically do not hunt immediately after consuming prey, it is reasonable to assume that between hunting bouts, the prey species have time to move. Therefore, the assumption in computing *E* from 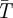 that the predator initially is randomly distributed with respect to the prey is not unreasonable. Although the movement data of three out of the four wolves showed evidence of anisotropic diffusion, only wolf 233 showed evidence of both anisotropic diffusion and bias towards seismic lines (see §3). Therefore, in order to compare the effects of the three different movement models on encounter rate, we estimated the coefficients for all the three models (no response, anisotropic diffusion and anisotropic diffusion + bias) from the wolf movement data for wolf 233 using the methods described in the electronic supplementary material, appendix.

We generated simulated landscapes with varying seismic line densities based on seismic line layers of west central Alberta mapped at a resolution of 5 m using Indian remote sensing satellite imagery [27]. A baseline seismic line density of 4.46 km km^−2^ was used to create landscapes with seismic line densities ranging from approximately 2 km km^−2^ to 9 km km^−2^ (electronic supplementary material, appendix figure S2). Prey were randomly placed in these landscapes at densities of 0.16, 0.5, 1, 1.5, 2, 2.5 and 3 prey km^−2^. We chose to include the density of 0.16 as it is similar to the lower range of density of common prey species in our study area [19]. We assumed wolves encountered a prey when they came within radius *r* = 100 m of the prey. In the model, this corresponds to a disc in the domain with radius *r* = 100 m, centred on the prey, with Dirichlet (*T* = 0) boundary conditions.

We evaluated the effect of wolf movement responses to seismic lines based on the first passage time solutions from two sets of simulations. First, for a fixed prey density of 0.16 prey km^−2^, we simulated the effects of the three different movement models outlined in table 1. Simulations were iterated 10 times at each seismic line density using a different distribution of randomly located prey. Encounter rates were plotted against seismic lines densities and visually inspected to assess the effects of movement responses to seismic line on encounter rates across a range of seismic line densities. Second, we assumed wolves moved according to the anisotropic diffusion model (table 1) and solved equation (2.1) for 10 replications of each prey density and seismic line density (i.e. six prey densities × 10 replications × 4 seismic line densities = 240 model solutions). We chose the anisotropic diffusion movement model because we found that it reflected the greatest effect of seismic lines on encounters with prey for our investigation. In each case, we used *T*(**x**) to compute the encounter rate using equations (2.2) and (2.3). We used the outputs of these simulations to evaluate a set of *a priori* candidate models (table 2) relating encounter rates to seismic line or prey densities or both using the nonlinear regression analysis package nls in R and comparing the fit of the models based on Akaike information criterion [28].

**Table 2.** Candidate models for encounter rate (*E*) of wolves as a function of prey density (*N*) and seismic line density (*S*).

model	explanation	form
*single variate models: seismic line density*		
A1	linear	*E* = *β*_0_ + *β*_1_*S*
A2	quadratic	*E* = *β*_0_ + *β*_1_*S* + *β*_2_*S*^2^
A3	exponential	*E* = *A*e^*bS*^
*single variate models: prey density*		
B1	directed search	*E* = *β*_1_*N*
B2	random search	*E* = *β*_1_*N*^2^
B3	combination of directed and random search, using a sum	*E* = *β*_1_*N* + *β*_2_*N*^2^
B4	combination of directed and random search using an intermediate power	*E* = *AN*^*b*^
*multivariate models*		
C1	no effect of seismic lines	*E* = *AN*^*b*^
C2	linear interaction between seismic lines and prey density	*E* = *AN*^*b*^ + *β*_1_*NS*
C3	nonlinear interaction between seismic lines and prey density (same power)	*E* = *AN*^*b*^ + *β*_1_*N*^*b*^*S*
C4	nonlinear interaction between seismic lines and prey density (different power)	*E* = *AN*^*b*_1_^ + *β*_1_*N*^*b*_2_^*S*

The functional forms of the candidate models (table 2) were proposed based on the results obtained earlier [3], which suggested that different underlying predator movement mechanisms lead to different relationships between encounter rate and prey density. McKenzie *et al.* [3] found that in one dimension, the encounter rate for predators undergoing only advective movement was a linear function of prey density. This is consistent with the assumption of a constant area searched per time made by Holling [1]. In contrast, McKenzie *et al.* [3] found that the encounter rate for predators undergoing only random movement was a quadratic function of prey density. Depending on the density of seismic lines, predators in our model undergo some combination of anisotropic diffusion along seismic lines and random searching off seismic lines via isotropic diffusion. Although the anisotropic diffusion model differs mathematically from the advective movement model, there is a similarity. Anisotropic diffusion biases movement in relation to both directions along the seismic line, whereas advection biases movement behaviour in relation to a single direction. Based on this similarity, we consider the anisotropic diffusion to describe a particular form of directed motion. Therefore, we proposed several model forms for the independent and combined effects of prey density and seismic lines that have a mechanistic basis for describing encounter rates when the search is a combination of directed and random motion by analogy with the earlier results [3].

### 2.4. The functional response

The functional response *f*(*N*) describes the *per capita* kill rate as a function of prey density *N*. Here, we investigate the potential variation in the functional response owing to increased predator mobility in the presence of seismic lines. To model the functional response, we use the Holling disc equation,2.7
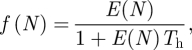
where the encounter rate *E* depends on prey density and *T*_h_ is the constant handling time. The handling time is defined by Holling to be the sum of the attack time (including evaluating, pursuing and catching the prey) and the handling time (including processing and consuming the prey). Traditionally, the encounter rate is assumed to be proportional to the prey density. Instead, we used the best multivariate model for encounter rate from table 2. Using the best model for encounter rate, we asked the question: how does the functional response change as the proportion of directed and random movement changes owing to increasing seismic line density? To answer this question, we compared the functional response in landscapes with seismic line densities of 0, 4.46 and 8.91 km km^−2^ by computing the ratio of the functional response at each seismic line density to that when there are no seismic lines present. If the ratio is 1, then seismic lines do not alter the kill rate. If the ratio is greater than 1, the presence of seismic lines leads to an increase in the kill rate. The larger the ratio, the larger the difference between the encounter rates in landscapes with and without seismic lines, and the stronger the effect of seismic lines. To see if the magnitude of the effect of seismic lines on the functional response depended on the handling time, we compared results assuming handling times for small-bodied (*T*_h_ = 10.6 h) and large-bodied (*T*_h_ = 20.4 h) prey [19].

## 3. Results

### 3.1. Wolf movements

**Figure 1.**
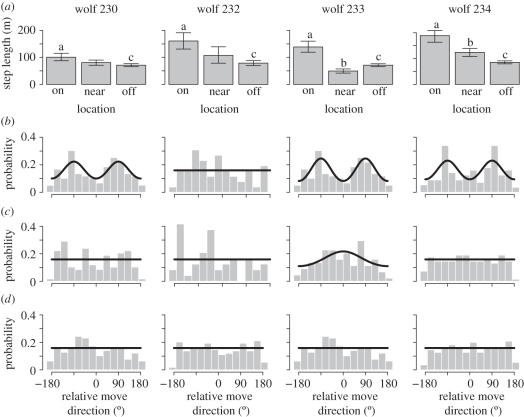
Mean step lengths and distributions of relative move directions of wolves calculated from 5 min GPS data. (*a*) Mean step lengths of wolves on, near and off seismic lines. Bars are 90% non-parametric bootstrapped confidence intervals. Relative move direction of wolves (*b*) on, (*c*) near, and (*d*) off seismic lines. Solid lines are the maximum-likelihood fit of the best model chosen using the PBLR test.

Visual inspection of the mean-squared displacements as a function of measurement time interval showed variability between wolves but exhibited approximately linear growth in the m.s.d. as a function of time, except at short time intervals (electronic supplementary material, appendix figure S3). When the 5 min move directions were constrained to include only those with step lengths of at least 55 m so as to remove errors (§2), we found statistically significant positive correlations in wolf movement directions with positive circular correlation coefficients of 0.20, 0.61, 0.33 and 0.43 for wolves 230, 232, 233 and 234. Movement patterns of wolves in landscapes with seismic lines were not consistent among individuals. All wolves had a longer mean step length on seismic lines than off seismic lines (figure 1*a*, *p* < 0.1). Wolves 230, 232 and 234 had a shorter mean step length off seismic lines than near seismic lines, while wolf 233 had a shorter mean step length near seismic lines. On seismic lines wolves 230, 233 and 234 had distributions of relative move directions that differed from the uniform distribution, with moves along seismic lines occurring more often than moves in other directions (figure 1*b*, *p* < 0.001). Wolf 232 moved randomly with respect to seismic lines. In contrast, near seismic lines, only wolf 233 had a non-uniform distribution of relative move directions (figure 1*c*, *p* < 0.001). When off seismic lines, all wolves had uniform distributions of relative move directions (figure 1*d*). We chose to use the movement parameters from the data for wolf 233 in our simulations because wolf 233 showed evidence of all the movement behaviours of interest (electronic supplementary material, appendix table S1). The maximum-likelihood fit of wolf 233 step lengths to the exponential probability density function yielded mean- squared displacement values of 0.017 km^2^ 5 min^−1^ off-lines and 0.043 km^2^ 5 min^−1^ on-lines (electronic supplementary material, appendix table S1) scaling up to 4.90 km^2^ d^−1^ off-lines and 12.4 km^2^ d^−1^ on-lines. The simulations of movement patterns yielded estimates for the off-line mean-squared displacement as 4.96 km^2^ d^−1^ and the on-line mean-squared displacement of 18.83 km^2^ d^−1^ for wolf 233, indicating that successive correlations in move direction have the effect of increasing the on-line mean-squared displacement per unit time by about 50 per cent. Other simulated mean-squared displacements for wolves were 5.86, 9.88 and 7.92 km^2^ d^−1^ (off-line) and 10.93, 24.08 and 32.26 km^2^ d^−1^ (on-line) for wolves 230, 232 and 234, respectively.

### 3.2. Model scenarios

**Figure 2.**
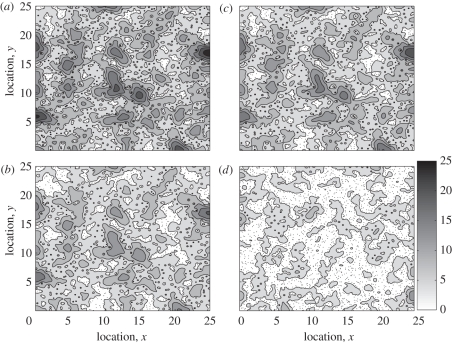
Example mean first passage time surfaces for the anisotropic diffusion model. (*a*–*c*) The prey density is 1.5 prey km^−2^ and the seismic line densities are 0, 4.46 and 8.91 km km^−2^, respectively. As seismic line density increases, the values of the mean first passage time surface decrease, leading to a decrease in the average mean first passage time, 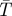. (*d*), There are no seismic lines, but prey density is increased to three prey km^−2^. Increasing prey density also leads to a decrease in the mean first passage time and the average mean first passage time.

Examples of the solution to mean first passage time equation (equation (2.1)) are shown in figure 2. For any point **x** = (*x*,*y*) in the domain, the value of the mean first passage time surface is the average time for a wolf starting at that location to encounter a prey item. Changes in prey density or seismic line density cause local differences in the mean first passage time surfaces. For example, surface height, the presence of peaks and the steepness of gradients differ between the example surfaces. These local differences translate into different average mean first passage time values, 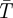 (see equation (2.2)), which we investigated further with statistical models.

**Figure 3.**
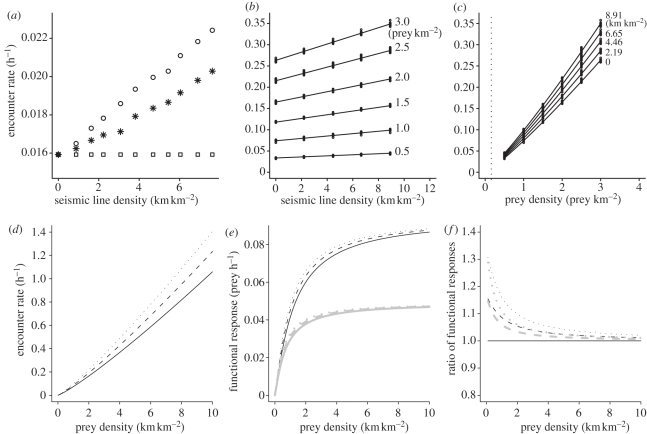
Simulated encounter rates (equations (2.1)–(2.3)) and the effect of seismic line density on the encounter rate and functional response. (*a*) Encounter rates for the different movement models as a function of seismic line density when prey density is fixed at 0.16 prey km^−2^ (squares, no response; circles, anisotropic diffusion; asterisks, anisotropic diffusion + bias). (*b*) Encounter rate at each prey density for the anisotropic diffusion model. Lines show the fit of the best model for encounter rate, which was the linear model in each case. (*c*) Encounter rate at each seismic line density for the anisotropic diffusion model. The dotted line indicates a density of 0.16 prey km^−2^ (compare with (*a*)). Lines show the fit of the best model for encounter rate, which was the power model in each case. (*d*) Predicted encounter rate for three seismic line densities. Encounter rate was predicted by the model C3: *E* = *AN*^*b*^ + *β*_1_ *N*^*b*^ *S*, where the coefficients are *A* = 7.43 × 10^− 2^, *b* = 1.15 and *β*_1_ = 2.74 × 10^−3^ (solid lines, no seismic lines; dashed lines 4.46 km km^−2^; dotted lines, 8.91 km km^−2^). (*e*) The functional response, assuming handling times for small-bodied (black lines) and large-bodied (grey lines) prey. (*f*) The ratio of functional responses in landscapes with and without seismic lines, assuming handling times for small- and large-bodied prey.

For a fixed prey density, encounter rate was constant when wolves did not alter their movement (no response model) with response to seismic lines (figure 3*a*). Encounter rates increased linearly with seismic line density when wolves followed a movement pattern resulting from faster movement on seismic lines and a tendency to continue along seismic lines once on them (anisotropic diffusion model). When an additional bias towards seismic lines when wolves were near seismic lines was included (anisotropic diffusion + bias model), encounter rates increased linearly but not as rapidly when compared with the model without the bias.

Given that seismic lines had the greatest effect on encounter rates under the anisotropic diffusion movement model, we examined the joint effects of seismic and prey densities for this movement mode (figure 3*b*,*c*). The model providing the best fit for the effects of seismic lines on encounter rate (electronic supplementary material, appendix table S2:A1) was consistently the linear model at all prey densities, but the slope of the relationship increased with prey density (figure 3*b*) indicating an interaction between seismic lines and prey density under this movement mode. A power model provided the best fit for relating encounter rate to prey density (electronic supplementary material, appendix, table S2:B4), where the constant scaling exponent (*b*), but variable *A* coefficient, also suggested a nonlinear interaction (electronic supplementary material, appendix table S3). Indeed, when encounter rate was modelled as a function of both prey and seismic line density, the best-fit model included a significant interaction term between seismic line and prey density (*p* < 0.001, electronic supplementary material, appendix table S3). The positive coefficient for the interaction term showed that seismic line density enhanced the rate at which the predators encountered prey.

### 3.3. The functional response

The observed increase in encounter rate due to increasing seismic line density translates into a functional response that increases more quickly to saturation (figure 3*d*,*e*). In all cases, the ratio of the functional responses in landscapes with and without seismic lines is greater than 1, meaning that the presence of seismic lines leads to an increase in kill rates (figure 3*f*). For handling times for both small-bodied and large-bodied prey, the ratio is larger at low prey densities than at higher prey densities. This suggests that the effect of seismic lines on the functional response is larger when prey density is low. In addition, across prey densities, the ratio of functional responses in landscapes with and without seismic lines is larger for small-bodied prey than for large-bodied prey. Therefore, the effect of seismic lines is more apparent when handling time is shorter.

## 4. Discussion

All four wolves in this study, as well as wolves in the boreal forests of Alberta [6], have demonstrated increased movement rates when travelling on seismic lines. Reduced debris and snow crusting on open seismic lines in winter compared with the forest may facilitate movement along the lines [6,29]. Three of the four wolves studied also exhibited a higher probability of continuing along seismic lines once on them. The propensity to remain on seismic lines is also reflected in habitat selection studies, where wolves were found to select seismic lines more than expected by chance [12]. The rapid movement and use of linear features have contributed to reports that wolves use them as travel routes [5,7,9,10,12]. The strong directional persistence of wolves while travelling on the seismic lines that we report may have been shaped, in part, by the straightness of seismic line across the landscape relative to other linear features like roads and trails [30]. Wolf 233 showed biased movement towards seismic lines when near them, which is the first quantification of such a bias, despite other reports of wolves changing their direction in order to move directly to adjacent compacted trails in nearby montane areas [7].

Incorporating these movement data into an advection–diffusion framework, we assessed the implications of varied movement strategies using the first passage time models on two movement components. We introduced anisotropy in the diffusion components of first passage models. At the same time, the possibility of movement bias towards seismic lines led us to introduce a bias term via advection. Both of these movement responses to seismic lines increased encounter rates with prey over when seismic lines were absent, consistent with the results of previous spatially explicit, individual-based models [31]. However, we also expected that the bias towards the seismic line, i.e. wolves being more likely to get on seismic lines than to leave them, would result in the highest encounter rates. This was not the case. In fact, although increased mobility was advantageous in covering more area to find prey, a movement bias towards seismic lines resulted in wolves finding fewer prey that were positioned away from the seismic lines. Unless prey are attracted to seismic lines or exist at very high densities (figure 3*c*), our results indicate that it would not be advantageous for predators to bias their movements towards seismic lines, explaining possibly why the bias was not commonly observed among the wolves we studied.

The purpose of fitting statistical encounter rate models to the first passage time simulation results was to assess how well the model parameters would reflect the underlying movement mechanisms being modelled. Under the anisotropic diffusion model, the statistical encounter rate models showed that wolf movement did not follow either a purely directed search (model B1) or a purely random search (model B2), even when no effect of seismic lines was included. Instead, the best model fit was intermediate, reflecting a mixture of both movement modes. This model exhibited a more than linear increase with respect to prey density, which is consistent with type III functional response. This is consistent with the results of McKenzie *et al.* [3], who showed that the encounter rate of randomly diffusing predators in one dimension was related to prey density by a power law.

Encounter rates increased linearly with seismic line density, but this increase was contingent on the distribution of prey with respect to the seismic lines [32]. Simulations were based on real landscapes from the study area. For this study area, seismic lines have been shown to be distributed randomly across the landscape, unlike some other linear features such as roads (E. H. Merrill 2005, unpublished data). Because prey were also assumed to be randomly distributed, the linear increase directly reflects that wolves spent more time in directed movement in landscapes with higher seismic line densities than when seismic lines were not present. If prey were to avoid seismic lines, as James & Stuart-Smith [12] have reported for caribou (*Rangifer tarandus*), encounter rates are expected to decline. However, as seismic line density increases, prey are less able to avoid seismic lines [33].

Our assumption that the ungulate prey did not move is a simplification that we needed to make to apply first passage time analysis to a complex spatial environment. Clearly, prey can move during the search period, although typically at a slower rate than the wolves, and this would have some effect on the results. However, it is unlikely that the wolves were following the prey on the seismic lines because their rates of movement were substantially higher than those of ungulate prey [13]. We assumed that all prey encountered were killed and that prey were encountered when wolves were within 100 m of the prey. While the success in killing a prey once detected can be highly variable, we simplified the model for the purpose of understanding the influence movements during search on functional response.

The presence of the interaction between prey density and seismic line density in the best-fit model indicates that the effect of prey density on encounter rate is modified by seismic line density. Therefore, it is not the effect of increasing seismic line density alone that leads to increased encounter rate, but the interaction between seismic line density and prey density. We interpret the positive coefficient of the interaction term to mean that increasing seismic line density will have a stronger effect on encounter rate at high prey densities than at low prey densities. Despite this interaction term leading to a larger increase owing to seismic lines in the magnitude of the encounter rate at high prey densities, using the Holling disc equation, we show that seismic lines have the greatest relative influence on potential kill rates in environments with low prey densities. This follows because predator mobility constrains searching success more acutely at low prey densities. As prey density increases, predators shift from being search limited to being handling time limited, and the benefit of increased mobility diminishes. However, even at high prey densities increased search efficiency may still alter predation rates when handling times are short. Although search time theoretically may not be the limiting process at high prey densities [1], wolves do not always invest the time in consuming the full prey and, at the extreme they have demonstrated surplus killing [34], which is consistent with short handling time. Additionally, in multi-prey systems, high search efficiency in environments with seismic lines may increase encounters with rare prey, that if more preferred or vulnerable, may result in dietary shifts [35], particularly for a coursing predator like the wolf, whose broad scale movements may homogenize spatial heterogeneity in prey [36]. Altered predation pressures within a prey community have implications of apparent competition and prey persistence ([37–39], see also [40]).

The approximately linear relationship between mean-squared displacement and measurement time interval (electronic supplementary material, appendix figure S3) indicates that a diffusion-based model may be appropriate for modelling the movement of wolves. However, the issue of positive correlations in the set of movement directions retained for analysis has implications for both the bootstrapping methods, which implicitly assume independence of data, and for the calculation of the diffusion coefficient, which assumes no persistence in movement direction. This is a difficult issue to deal with in a satisfactory manner. One approach for reducing correlations in move directions is to subsample data by taking it over less frequent time intervals [17]. However, we chose not to use this subsampling approach because it would have removed the important detailed information required to see how wolves move in relation to roads over short spatial scales. A second approach is to rescale the estimate of the diffusion coefficient based on the simulations of wolf movement on- and off-lines. This would have left the off-line diffusion coefficient the same but would have increased the on-line diffusion coefficient by approximately 50 per cent. An alternative method for correcting the diffusion coefficient, which leads to similar results, is based on the work of Patlak (see appendix C of Moorcroft & Lewis [17]). This attempts to approximately correct for persistence in movement direction by rescaling the diffusion coefficient by 1/(1 − *ψ*), where *ψ* is the mean cosine of the turning angle, and the turning angle is the difference in consecutive movement directions. We measured that the quantity *ψ* averaged 0.18 off seismic lines and 0.43 on seismic lines, suggesting that correlations could make the diffusion coefficient 22 per cent higher off seismic lines and 75 per cent higher on seismic lines. While Patlak's approach can deal with spatial heterogeneity, it has not yet been extended to the case of anisotropy, which is a central element in our model. Regardless, we also chose not use either of these methods to rescale the diffusion coefficient, noting that the variation calculated between the different wolves is larger than correction term that such rescaling would entail. However, had we made such a rescaling, the overall first passage times would be somewhat reduced, and the effect of seismic lines on first passage times would be enhanced. A third mathematically interesting approach to deal with correlations is to use a non-diffusive model, based a velocity jump process where there is persistence in movement direction. This non-diffusive *transport equation* framework has only just recently been extended to deal with anisotropic movement patterns [41] and the connection with first passage time analysis has not yet been developed. Finally, a full analysis of correlations must also include autocorrelations over periods longer than a single time step, which have the potential to further increase the mean-squared displacement per unit time (electronic supplementary material, appendix figure S3), as well as interactions from measured correlations with GPS errors. We suggest that these are important avenues for future research.

Our understanding of the influence of predator response to spatial heterogeneity on search behaviours and its implications for predator–prey interactions is now emerging [3,42–45]. Because details on animal movements are now readily available with GPS technology [46], it may be possible to quantify distinct modes of movement [47–50] even if an understanding of motivation for the movement behaviours is less clear [51]. In this paper, we have shown that a new alternative model structure for the encounter rate component of the functional response is appropriate when predators alter their movement in response to landscape heterogeneity. We illustrate the point for wolves in real landscapes where seismic line densities alter the directional component of search. In terms of conservation, our results indicate that increasing seismic line density or other linear features associated with land development that affects wolf movement will have a relatively large impact on single prey systems when the prey is at risk (i.e. low density) and small (i.e. small handling times) or on multi-prey systems where there is preference for the rare species.
